# Supplementary material for: How are public engagement health festivals evaluated? A systematic review with narrative synthesis
Source: PLoS One. 2022 Aug 23;17(8):e0267158. doi: 10.1371/journal.pone.0267158 (PMC9398006; doi:10.1371/journal.pone.0267158)
Supplement: S1 File — (PDF) [file pone.0267158.s002.pdf]

REGISTRY OF SYSTEMATIC REVIEWS/META-ANALYSES DETAILS

Registration Date

October 22, 2020 15:03

Review Registry Unique Identifying Number

reviewregistry1021

Title

How are health-related public engagement festivals evaluated? A systematic review with narrative synthesis.

Key Review Question and Objectives

We intend to:
1) Complete a systematic review to synthesise the evidence from health-related public engagement festival evaluations reports published in peer-reviewed journals.
2) Using narrative synthesis, review the methods and outcomes of health-related public engagement festival evaluations up to now.
3) Compare synthesis findings to the QMUL toolkit.
4) Reflect on improvements to public engagement evaluations.

Primary Research Question: What methods and outcomes are reported in published evaluations of health-related public engagement festivals?

Secondary Research Question: How do the evaluation methods used in these reports compare to those outlined in the Queen Mary University of London Public Engagement Toolkit (Queen Mary University of London. Parts 1 and 2: The Toolkit. 2018.) and (Queen Mary University of London. Part 3: Evaluation Tools. 2018.)

Lay Summary (please do not just paste your abstract. Please summarise your research, written in a way the public can understand)

Science festivals are growing in popularity as a means of disseminating and engaging the public in research. Evaluating public engagement events for their design, delivery and impact is important for understanding the effectiveness of the engagement event. The Public Engagement Evaluation Toolkit from Queen Mary University of London (QMUL) delivers tools and guidance about evaluating public engagement, which aims to improve public engagement evaluations. This systematic review will therefore review the effect and implementation of health-related public engagement evaluations using narrative synthesis and compare the findings to the QMUL guidance. Consideration will be given to how festival evaluation could be improved.

Does this Systematic Review include a Meta-Analysis?

No

Primary Investigator

Lucy Selman

Other Investigator(s)

Suannah Martin

Additional Investigators

Charlotte Chamberlain

Participating Institution(s)

University of Bristol, Bristol, UK
Contact Details for Public/Scientific Enquiries (include postal address)
Bristol Medical School (Population Health Sciences), Faculty of Health Sciences, University of Bristol, BS8 2PS
Primary Email
lucy.selman@bristol.ac.uk
Secondary email
yg19337@bristol.ac.uk
Telephone number for enquiries
+44 (0)117 928 9000

Countries involved
England

Funding
University of Bristol

Search Strategy
"public engagement"

AND
Festival OR event

AND
Science OR research OR health

Information Sources (describe all information sources (e.g., databases with dates of coverage, contact with study authors to identify additional studies) in the search and dates you will or last searched)
The following databases will be searched for English language studies published from the year 2000 up to present: MEDLINE on OvidSP, Web of Science - core collection, Embase, and CINAHL.
We will also hand-search the reference lists and complete citation tracking of eligible papers.

Inclusion Criteria
Evaluation of a single or multi-year public engagement festival. All empirical study designs will be included. Art, culture or science festivals which have an identified health-related activity and where the evaluation is of this element will be included. Studies including families or children and adults, will be included if the adult data can be separated or isolated.
Studies should self-identify as a 'festival'.
Only studies which are published from the year 2000 to current will be included, since most public engagement festivals have emerged in the last twenty years. Only studies published in the English language will be included.

Exclusion Criteria
Studies evaluating both a festival and other public engagement formats or several different festivals, where evaluation data of the health-related festival cannot be separated or isolated will be excluded. Studies will be excluded if they use festivals to recruit participants into studies or use festival attendees for involvement in policy,

research or service planning and prioritisation rather than public engagement i.e. those festivals with no two-way dialogue element. Studies which use festivals purposefully as health interventions rather than for public engagement purposes will also be excluded.
We will exclude festivals which evaluate the impact only on children or student and teacher participants.
Festivals which are only religious, art or music-based i.e. have no health-related science or research remit, will be excluded.

Condition, disease or problem being studied

The evaluation of public engagement health festivals.

Definition of 'Public Engagement'
Two-way dialogue between scientists and members of the general-public. We focus here on engagement in relation to health-related science and research, including medicine and applied health.

Definition of 'festival': A live event which engages the public in health-related science. The event is transient, provides a brief and concentrated focus on the topic and takes places in a specific place or region.

Patients/Participants/Population

Adult members of the general public i.e. citizens who are non-specialists and not in academia or teaching, who are attending / have attended the public engagement festival.

Intervention(s) or Exposure(s)

Health-related public engagement festivals.

Control or Comparator(s)

Not applicable.

Primary Outcomes

The review will identify documented outcomes e.g. awareness raising, and outcome measures employed e.g. attendance, to evaluate public engagement festivals. Main outcomes which may not have been explicitly stated, but were measured and reported e.g. reach, satisfaction, impact, will also be described.
The review will also describe the evaluation methods used, for instance pre-post questionnaires, frequency counts or interviews to evaluate public engagement health festivals.

Secondary Outcome(s)

Not applicable

Data extraction (selection and coding)

Titles and/or abstracts of records retrieved using the search strategy will be screened independently by SM to identify study reports which potentially meet the inclusion criteria. The full text of remaining records will be retrieved and independently assessed for eligibility by SM. A random sample of 10% will be screened by LS or CC to check for consistency in application of the eligibility criteria. Any disagreement over study eligibility will be resolved through discussion between

the three researchers. A data extraction report will be developed and piloted during the screening stage. Data will be extracted according to the following headings: First authors' name, title, year of publication, aim of festival, aim of the evaluation, location of study, internal/ external evaluation, evaluators e.g. volunteers or scientists, sample size, timing of evaluation, evaluation methods, evaluation outcomes, evaluation conclusions.
Additional data will be extracted and appraised against the QMUL toolkit under the headings taken from the full evaluation planning template (Queen Mary University of London. Parts 1 and 2: The Toolkit. 2018):
Design: Have they evaluated the design of their public engagement? Does the design follow good practice underpinned by sound ethics? (The additional four questions under this sub-heading, as per the toolkit, will be used, if applicable). How well do they know the context they are working in, and have they adapted the design of their activities to this context? (The additional three questions under this sub-heading as per the toolkit will be used if applicable).
Delivery/ Outputs: What immediate outputs did they want to deliver from engagement? How would they have known they delivered these outputs (what indicators did they identify)? What tool(s) did they use to track their progress?).
Impacts: What benefits or "impacts" did they want to achieve from engagement? How did they know they achieved these impacts (what indicators did they identify)? What tool(s) did they use to track their progress?.

SM will extract data, and LS and CC will independently review 20% of the data extraction, to check for refinement or omission of data. Any discrepancies will be identified and resolved through discussion.

Risk of bias (quality) assessment

The quality of included studies will be assessed by SM using the Mixed Methods Appraisal Tool (MMAT) – Version 2018 (Hong et al. Mixed Methods Appraisal Tool (MMAT) version 2018 User Guide. Retrieved on 30/06/2020 available from: http://mixedmethodsappraisaltoolpublic.pbworks.com/w/file/fetch/127916259/MMAT_2018_criteria-manual_2018-08-01_ENG.pdf). The MMAT is a validated critical appraisal tool and is appropriate for this systematic review since it allows for the appraisal of the methodological quality of qualitative, quantitative, mixed-methods, randomised controlled and non-randomised studies. LS and CC will review 20% of the quality assessment for consistency and judgement. Any disagreements between review authors over the quality of the eligible primary study will be resolved through discussion. As recommended in the MMAT user guide, studies will not be excluded on the basis of their quality, but, the narrative synthesis will reflect and include discussion on the quality of the included studies.

Data Synthesis Strategy

We will conduct a narrative synthesis of the studies, following the framework stages proposed by Popay et al (2006) i.e.

1. Developing a theoretical model of how the intervention works, why and for whom
2. Developing a preliminary synthesis
3. Exploring relationships in the data
4. Assessing the robustness of the synthesis product.

SM will lead the synthesis, with regular meetings with LS and CC to review emerging findings and patterns. The narrative synthesis will be structured around the methods used for public engagement festival evaluation and the outcomes of the study

evaluation. The synthesis findings will be qualitatively compared to the Queen Mary University of London Public Engagement Toolkit (2018). We will also reflect on improvements to public engagement festival evaluations.

Analysis of subgroups or subsets

This is a narrative synthesis and therefore it is not possible to specify any particular subgroups for analysis in advance.

Dissemination plan

A manuscript will be prepared for submission to a peer-reviewed journal.

Stage of Review at Time of Submission

Protocol Written

Post Review Results (once available)

User
