# Supplementary material for: How are public engagement health festivals evaluated? A systematic review with narrative synthesis
Source: PLoS One. 2022 Aug 23;17(8):e0267158. doi: 10.1371/journal.pone.0267158 (PMC9398006; doi:10.1371/journal.pone.0267158)
Supplement: S1 Table — (DOCX) [file pone.0267158.s003.docx]

**Table S1: Search strategy**

All searches were restricted to English language papers only and from year 2000 – Current.

| **Database** | **Search strategy** |
| --- | --- |
| **OvidSP MEDLINE and**  **Embase** | 1. Public engagement.mp 2. Festival.mp 3. Event.mp 4. Science.mp 5. Research.mp 6. Health.mp 7. 2 or 3 8. 4 or 5 or 6 9. 1 and 7 and 8 10. Limit 9 to (English language and yr=”2000-Current”)   .mp = multiple places |
| **Web of Science – core collection** | #1 ALL=(“public engagement”)  #2 ALL= (health)  #3 ALL=(science)  #4 ALL=(research)  #5 ALL=(event)  #6 ALL=(festival)  #7 #4 OR #3 OR #2  #8 #5 OR #6  #9 #8 AND #7 AND #1  *Timespan=2000-2020*  (ALL=) = All Fields |
| **CINAHL** | S1. TX public engagement  S2. TX festival  S3. TX event  S4. TX science  S5. TX health  S6. TX research  S7. (S1 AND (S2 OR S3) AND (S4 OR S5 OR S6))  TX = All Text |
