# Supplementary material for: How are public engagement health festivals evaluated? A systematic review with narrative synthesis
Source: PLoS One. 2022 Aug 23;17(8):e0267158. doi: 10.1371/journal.pone.0267158 (PMC9398006; doi:10.1371/journal.pone.0267158)
Supplement: S2 Table — (DOCX) [file pone.0267158.s004.docx]

**Table S2: Queen Mary University of London (QMUL) Toolkit Headings***

| **DESIGN** | **DELIVERY / OUTPUTS** | **IMPACT** |
| --- | --- | --- |
| Have they evaluated the design of their public engagement? | What immediate outputs did they want to deliver from engagement? | What benefits or "impacts" did they want to achieve from engagement? |
| Does the design follow good practice, underpinned by sound ethics? i.e.   - Have they systematically identified relevant publics (and stakeholders)? - Did they understand the expectations and specific benefits each group was likely to derive from engagement? - Did they identify and make contingencies for any risks & assumptions? - Did they test their activities and seek feedback from relevant publics? | How would they have known they delivered these outputs (identify indicators)? | What tool(s) did they use to track their progress? |
| How well did they know the context they were working in and did they adapt the design of their activities to this context? i.e.   - Did their proposed engagement activities match the interest and needs of their target publics and their social and cultural context? - Was there experience of engagement and existing trust between members of the research community and publics? - Did they have sufficient resources and support for engagement in this particular context e.g. professional facilitation, event planning etc.? | How would they have known they'd achieved these impacts (identify indicator(s)? | What tool(s) did they use to track their progress?” |

***** Data were extracted into the journal citation report against every heading from the QMUL toolkit. Headings were taken directly from the toolkit (Queen Mary University of London, 2018) [25]
