# Supplementary material for: How are public engagement health festivals evaluated? A systematic review with narrative synthesis
Source: PLoS One. 2022 Aug 23;17(8):e0267158. doi: 10.1371/journal.pone.0267158 (PMC9398006; doi:10.1371/journal.pone.0267158)
Supplement: S3 Table — (DOCX) [file pone.0267158.s005.docx]

**Table S3: Study sample size, response rate and evaluation method**

| **Study reference number** | **Size of sample evaluated** | **Response rate** | **Primary evaluation method** | **Researchers’ team’s relationship to festival** | **Evaluators’ relationship to researchers** | **Festival Aims** |
| --- | --- | --- | --- | --- | --- | --- |
| **1** | 324 | 43.9% | Post-event questionnaire | Independent | Connected | Specific |
| **2** | 176 | 42% | Post-event questionnaire | Unclear | Unclear | Specific |
| **3** | 37 | 100% | Activity output/ observation/ recording Q’s | Independent | Connected | Specific |
| **4** | 183 (event attendees)  948 (control group)  34, 26 (pre- and post-panel event) | 79.2% (event audience)  50.3% (control group)  94.1%, 100% (pre- and post- panel event) | Survey with control group | Independent | Independent | Specific |
| **5** | 38 | 68% | Post-event questionnaire | Connected | Connected | Broad |
| **6** | 661 | 7% | Post-event questionnaire | Independent | Independent | Specific |
| **7** | 121 | 71% | Post-event questionnaire | Independent | Connected | Broad |
| **8** | 415 | 9.7% - 63.6%. | Pre-post questionnaire | Independent | Unclear | Specific |
