# Supplementary material for: How are public engagement health festivals evaluated? A systematic review with narrative synthesis
Source: PLoS One. 2022 Aug 23;17(8):e0267158. doi: 10.1371/journal.pone.0267158 (PMC9398006; doi:10.1371/journal.pone.0267158)
Supplement: S4 Table — (DOCX) [file pone.0267158.s006.docx]

**Table S4:** Quality Assessment using the Mixed-Methods Appraisal Tool (MMAT)*

| **Study Ref** | **S1** | **S2** | **1.1** | **1.2** | **1.3** | **1.4** | **1.5** | **4.1** | **4.2** | **4.3** | **4.4** | **4.5** | **5.1** | **5.2** | **5.3** | **5.4** | **5.5** |
| --- | --- | --- | --- | --- | --- | --- | --- | --- | --- | --- | --- | --- | --- | --- | --- | --- | --- |
|  |  |  | **Qualitative** | | | | | **Quantitative descriptive** | | | | | **Mixed-methods** | | | | |
| **1** | Yes | Yes | Yes | Yes | Yes | Yes | Yes | Yes | Unclear | Yes | Unclear | Yes | Unclear | Yes | Yes | Yes | Unclear |
| **2** | Yes | Yes | Yes | Yes | Yes | Yes | Yes | Yes | Yes | Yes | Unclear | Yes | Unclear | Yes | Yes | Yes | Yes |
| **3** | Yes | Yes | Yes | Yes | Yes | Yes | Yes | Yes | Yes | Yes | Yes | Yes | Yes | Yes | Yes | Yes | Yes |
| **4** | Yes | Yes | n/a | n/a | n/a | n/a | n/a | Yes | Yes | Yes | Yes | Yes | n/a | n/a | n/a | n/a | n/a |
| **5** | No | Unclear | Unclear | Unclear | Unclear | Unclear | Unclear | Unclear | Yes | Yes | Unclear | Yes | Unclear | Yes | Yes | Unclear | Unclear |
| **6** | Yes | Yes | Yes | Yes | Yes | Yes | Yes | Yes | Yes | Yes | Yes | Yes | Yes | Yes | Yes | Yes | Yes |
| **7** | No | Unclear | Unclear | Yes | Unclear | Yes | Unclear | Unclear | Yes | Yes | Unclear | Unclear | Unclear | Yes | Yes | Unclear | Unclear |
| **8** | Yes | Yes | Yes | Yes | Yes | Yes | Yes | Yes | Yes | Yes | Yes | Yes | Yes | Yes | Yes | Yes | Yes |

*Headings taken directly from the MMAT are listed below:

**“Screening questions (for all types)**

S1. Are there clear research questions?

S2. Do the collected data allow to address the research questions?

**1. Qualitative**

1.1. Is the qualitative approach appropriate to answer the research question?

1.2. Are the qualitative data collection methods adequate to address the research question?

1.3. Are the findings adequately derived from the data?

1.4. Is the interpretation of results sufficiently substantiated by data?

1.5. Is there coherence between qualitative data sources, collection, analysis and interpretation?

**4. Quantitative descriptive**

4.1. Is the sampling strategy relevant to address the research question?

4.2. Is the sample representative of the target population?

4.3. Are the measurements appropriate?

4.4. Is the risk of nonresponse bias low?

4.5. Is the statistical analysis appropriate to answer the research question?

**5. Mixed-methods**

5.1. Is there an adequate rationale for using a mixed methods design to address the research question?

5.2. Are the different components of the study effectively integrated to answer the research question?

5.3. Are the outputs of the integration of qualitative and quantitative components adequately interpreted?

5.4. Are divergences and inconsistencies between quantitative and qualitative results adequately addressed?

5.5. Do the different components of the study adhere to the quality criteria of each tradition of the methods involved?”[32]
